# External validation of type 2 diabetes computer simulation models: definitions, approaches, implications and room for improvement—a protocol for a systematic review

**DOI:** 10.1186/s13643-017-0664-7

**Published:** 2017-12-29

**Authors:** Katherine Ogurtsova, Thomas L. Heise, Ute Linnenkamp, Charalabos-Markos Dintsios, Stefan K. Lhachimi, Andrea Icks

**Affiliations:** 1 Institute for Health Services Research and Health Economics, German Diabetes Center (DDZ), Leibniz Center for Diabetes Research at Heinrich Heine University, Auf’m Hennekamp 65, 40225 Düsseldorf, Germany; 2 Institute for Public Health and Nursing Research—IPP, Health Sciences Bremen, University of Bremen, Bremen, Germany; 3 Research Group for Evidence-Based Public Health, Leibniz Institute for Prevention Research and Epidemiology—BIPS, Bremen, Germany; 4 Institute for Health Services Research and Health Economics, Centre for Health and Society, Faculty of Medicine, Heinrich-Heine University Düsseldorf, Düsseldorf, Germany; 5 German Center for Diabetes Research (DZD), Neuherberg, Germany

**Keywords:** Type 2 diabetes mellitus, Diabetes models, Simulation, External validation, Systematic review

## Abstract

**Background:**

Type 2 diabetes mellitus (T2DM), a highly prevalent chronic disease, puts a large burden on individual health and health care systems. Computer simulation models, used to evaluate the clinical and economic effectiveness of various interventions to handle T2DM, have become a well-established tool in diabetes research. Despite the broad consensus about the general importance of validation, especially external validation, as a crucial instrument of assessing and controlling for the quality of these models, there are no systematic reviews comparing such validation of diabetes models. As a result, the main objectives of this systematic review are to identify and appraise the different approaches used for the external validation of existing models covering the development and progression of T2DM.

**Methods:**

We will perform adapted searches by applying respective search strategies to identify suitable studies from 14 electronic databases. Retrieved study records will be included or excluded based on predefined eligibility criteria as defined in this protocol. Among others, a publication filter will exclude studies published before 1995. We will run abstract and full text screenings and then extract data from all selected studies by filling in a predefined data extraction spreadsheet. We will undertake a descriptive, narrative synthesis of findings to address the study objectives. We will pay special attention to aspects of quality of these models in regard to the external validation based upon ISPOR and ADA recommendations as well as Mount Hood Challenge reports. All critical stages within the screening, data extraction and synthesis processes will be conducted by at least two authors. This protocol adheres to PRISMA and PRISMA-P standards.

**Discussion:**

The proposed systematic review will provide a broad overview of the current practice in the external validation of models with respect to T2DM incidence and progression in humans built on simulation techniques.

**Systematic review registration:**

PROSPERO CRD42017069983.

**Electronic supplementary material:**

The online version of this article (10.1186/s13643-017-0664-7) contains supplementary material, which is available to authorized users.

## Background

Diabetes mellitus (DM), characterised by increased blood glucose concentration, is a major health care challenge: more than 415 million adults (20–79 years old), or 8.8% in the given age group, were living with this chronic condition in 2015 according to the latest estimates of the International Diabetes Federation (IDF). This figure, even by conservative projections, will reach 642 million in 2040 [1]. While there is a spectrum of metabolic disorders under the label ‘diabetes’, the majority of cases (90 to 95% [2]) may be classified as type 2 diabetes mellitus (T2DM).

T2DM is a chronic condition that may often remain undiagnosed over several years. Medical conditions related to T2DM, such as retinopathy, often emerge before the time of the clinical diagnosis of T2DM [3, 4]. Moreover, final stage complications such as blindness, limb amputation, renal failure, stroke or myocardial infraction result in high health care expenditures and loss of healthy life years that put an unstringed burden on a patient’s health and the health care system in general [5].

Computer simulation models are often employed to evaluate the clinical and economic effectiveness of various interventions that are considered for implementation or have already been implemented in a health care system [6–8]. Using computer simulation models allows addressing characteristics of different patient populations extensively under multiple independent conditions and treatments. Furthermore, models can simulate health outcomes over periods of more than 5–10 years, which is useful for chronic conditions when considering the long-term clinical and economic impact of different intervention scenarios [9]. Modelling assists clinicians or policy makers in the decision making process by synthesizing the existing evidence base in a transparent way with regard to complexity, variability and uncertainty of health and disease progression [9].

Diabetes modelling is a relatively recent technique in health economic analysis. The first publication of a fully integrated (including the full range of complications) economic model of type 1 diabetes mellitus (T1DM) was published in 1996 [10]. As shown in systematic reviews regarding this topic [11], the first influential work on T2DM modelling was the article ‘Model of Complications of NIDDM: I. Model construction and assumptions’ by Eastman et al. published in 1997 [12]. Now, computer simulation is recognized as a well-established method to harmonize and personalize the abundance of evidence on long-term effects of diabetes, which helps to answer ‘what if’ questions about treatment effects. Researchers intend using the models in the analysis of clinical and cost-effectiveness of different, mostly pharmacological, interventions [11, 13, 14].

Therefore, it is of utmost importance that there is confidence in the models to provide an accurate reflection of disease progression in real life [15, 16]. This is particularly true for chronic diseases such as T2DM, which develop over a long period of time and are associated with significant morbidity and mortality and a substantial burden to the health care system and to society.

Several ways of assessing and controlling for the quality of these models are in use, in which validation is a crucial part [15–17]. According to the recommendations given in the report of the International Society for Pharmacoeconomics and Outcomes Research (ISPOR) Good Research Practice Task Force, validation of models is categorized in three main groups: internal validation, between-model validation, and external validation [18]. The American Diabetes Association (ADA) released guidelines [9] to standardize the description and validation of diabetes models. They defined criteria that, if followed, would build confidence for a model to accurately perform its intended function: the steps that can be taken by model developers to ensure that others can reproduce the results and build confidence that the models are accurate, useful and reliable.

‘External validation’ refers to the ability of the model to accurately predict or replicate the results of studies that were not used to build the model [18, 19]. This ability of replicated external studies is appreciated as a model’s intended purpose [9]. Eddy et al. [19] gave the best practices of a formal process to conduct external validation. They suggested that modellers should make a description of the external validation process and its results available on request. Moreover, they should identify parts of the model that cannot be validated given a lack of suitable sources and describe how the related uncertainty was addressed.

The Mount Hood Challenge meetings are organized to promote validity and reliability in diabetes modelling [16]. The first meeting of health economic simulation modelling groups focusing on diabetes took place in 1999 and challenged only two simulation models [20]. Several of the later meetings were focusing on the external validation of models but only for models developed by researchers attending the meeting [15, 16]. However, the organisers stated that there was still no clear consensus on what precisely model validation means [15]: appropriate statistical approaches should be defined to assess correlation between model and clinical trial outcomes, and limits could be predefined for model accuracy and precision.

Several systematic literature reviews comparing and assessing the quality of diabetes models are available. In 2010, two reviews included DM models published before 2008 and focused on health-economic aspects of diabetes models, specifically, their use for pharmacological treatment evaluations [11, 13]. Three years later, a review by Charokopou et al. updated results by including studies published between 2008 and 2013 [14]. Moreover, a systematic health economic assessment of three models (the US Centers for Disease Control and Prevention (CDC-RTI) Diabetes Cost-effectiveness Model [21], the Quintiles IMS CORE Diabetes Model (CORE) [22], and the Archimedes model [23]) was conducted by Becker et al. in 2011 [24]. In 2016, Henriksson et al. [25] focused on economic models of T1DM and provided an overview of the characteristics and capabilities of available models. Also, Kirsch published a systematic review of Markov models evaluating multicomponent disease management programs for DM in 2015 [26].

In four of six literature reviews (Yi et al. [13], Becker et al. [24], Tarride et al. [11], Charokopou et al. [14]), the authors evaluated whether internal and external validation of the models was reported. Only in one review [24] did the authors appraise the validation approaches by applying the criteria to assess a models’ quality recommended by the panel of the ADA [9]. Becker et al. [24] found that while there were reports on extensive validations for all of the assessed models, results were not directly comparable due to different outcomes, studies or populations used. By focusing on the use of DM models in practice, these reviews may not have identified all published models in the field of T2DM per se and may not have identified all evidence associated with the validation of the identified models.

Despite the broad consensus about the general importance of the validation of models presented in the literature, there are to our knowledge no systematic reviews comparing the practice of validating diabetes models. Such a systematic review might serve to understand, improve and critically appraise current practices, in particular, in the context of the external validation.

The lack of extensive reviews in regards to validation of the models can be explained by the specific challenges faced in the field. The models are diverse in their structure and the underlying assumptions and use of inhomogeneous methods to conduct the validation and to report their results and respective methodology. Furthermore, most of the diabetes models are built on the same data sources (e.g. The United Kingdom Prospective Diabetes Study (UKPDS)) [11, 14, 25], or the availability of data sources is limited.

### Study objective and rationale

The main objective of this systematic literature review is to identify and appraise the quality of approaches that are being used for the external validation of existing computer models covering the development and progression of T2DM in human populations.

We will review identified models with regard to the validation efforts to provide an overview of current practices reported in the literature and to find out if these practices are in line with the recommendations given elsewhere [9, 15–17].

We will summarise the findings of the studies by each model identified. The information from articles and reports on each model will be aggregated and compared with other models.

## Methods

### Protocol

This protocol adheres to the Preferred Reporting Items in Systematic Reviews and Meta-analyses (PRISMA) statement [27] and PRISMA for systematic review protocols (PRISMA-P) statement [28, 29]. The PRISMA-P checklist is given in Table 3 in the Appendix. The protocol is registered in the International Prospective Register of Systematic Reviews (PROSPERO) CRD42017069983.

### Eligibility criteria

We will include studies reporting and describing the use of a computer simulation model with the characteristics given in Table 1.

**Table 1:** Eligibility criteria in study selection

Criteria	Inclusion	Exclusion
Model structure	• The model focuses on the incidence and/or progression of T2DM;	• The model does not describe the onset and the development of T2DM but focuses on a partial aspect of the disease; or
	• The model has an explicit disease state structure: {{healthy} or {IGT}(Impaired Glucose Tolerance) or {IFG} (Impaired Fasting Glucose) or {pre-diabetes or {high-HbA1c}} and {{T2DM} or {T2DM with complication(s)}}. IGT, IFG, high-HbA1c and diabetes should be defined according to ADA [34] or WHO [35] criteria;	
	• The model is based on a real-life population: a tool must be able to model different populations that vary in age and/or gender structure. The starting population may comprise healthy individual (cohorts) and/or individuals (cohorts) in advanced stages of T2DM with/or without complications;	• The model is covering only T1DM.
	• The model has employed a projection: a tool must be able to appraise changes in the health of the population over time (both short- (1 to 10 years) and long-term (> 10 years) projections); and	
	• The model has employed explicit risk factors (for example, comorbidities, BMI, hypertension, level of total cholesterol, socio-economic status, education, ethnicity, smoking, etc.): risk factors and their impact on the disease progression are explicitly displayed in the model at every time period and for every individual or subgroup units.	
Methods/techniques	• The model is based on simulation techniques; and	• The modelling does not incorporate simulation techniques.
	• The simulation is built on individual units representing an organ, a person or a cohort.	
Study type	• The study is based on single and/or multiple data sets.	
Type of publication	• Publications in peer-reviewed journals which can be retrieved through our search approach; and	• Editorials, comments, newspaper articles and other forms of popular media;
	• Studies, to which full publication is available.	• Conference abstracts; or
		• Studies, to which no full publication is available.
Date	• The study has been published in 1995 or later.	• The study has been published before 1995.
Language	• No restrictions on language; and	• Studies with no translation into English or German possible.
	• Studies written not in English or German will be labelled as ‘other languages’. In order to obtain further information or full translations in English, we will contact corresponding authors.	

We will exclude studies published before 1995 from our review because computer simulation of diabetes is a relatively new concept. Two previous systematic literature reviews [25, 26] also restricted the time frame of their searches to the year 1995: the authors justified the limitation by the fact that the first two relevant publications were published in 1996 [10] and in 1997 [12], respectively.

### Information sources

We will search the following literature databases: MEDLINE (via NLM, PubMed), CENTRAL (via Wiley), EMBASE (via Ovid SP), EconLit (via EBSCOhost), Web of Science (via Thomson Reuters), PsycINFO (via Ovid SP), Scopus (via Elsevier) and NHS Economic Evaluation Database (NHS EED) (via Wiley).

To identify potential existing systematic reviews with regard to our research topic, we will also search the Cochrane Database of Systematic Reviews (CDSR) (via Wiley) and the Database of Abstracts of Reviews of Effects (DARE) (via Wiley).

We will also include grey literature databases in our search: ProQuest Dissertations & Theses Database (PQDT) (via ProQuest), System for Information on Grey Literature in Europe (OpenGrey) (via INIST/CNRS), The Directory of Open Access Repositories (OpenDOAR) (via CRC) and CINAHL (via EBSCOhost). In PQDT, the search will be limited to titles only since this database contains very unspecific abstracts. In OpenGrey and OpenDOAR, the results will be limited to the first 100 hits due to the Google Custom Search-based procedure. The grey literature will be concerned to widen the search scope and to capture models that were not highlighted in previous systematic literature reviews.

Besides references identified through databases and systematic reviews, we will include models published in the Mount Hood Challenge meetings proceedings [15, 16].

### Search strategy

We composed the ‘primary’ bibliographic search strategy according to MEDLINE search rules, which will be later translated to other database syntaxes. We have applied the search strategy to the relevant references of studies found in the previous literature reviews and/or known in the research field to check for the search strategy’s sensitivity. This primary search strategy has been peer-reviewed using a structured checklist given in the PRESS Peer Review of Electronic Search Strategy: 2015 Guideline Statement [30]. The peer review assessment is given in Additional file 1.

The details of this search strategy are given in Table 2.

**Table 2:** Search strategy for MEDLINE (via PubMed)

Line	Search syntax	*N* of hits (2017/08/09)
Subject of interest: DM		
1	((((((((((“Diabetes Mellitus” [MeSH Terms]) OR diabet* [Title/Abstract]) OR T2DM [Title/Abstract]))	577,871
Analyses of interest: computer simulation or Markov process		
2	((((((((((markov [Title/Abstract]) OR “monte carlo” [Title/Abstract]) OR “montecarlo” [Title/Abstract]) OR stochastic [Title/Abstract]) OR deterministic [Title/Abstract]) OR “discrete event” [Title/Abstract]) OR “agent based” [Title/Abstract]) OR “agentbased” [Title/Abstract])) OR “Computer Simulation” [MeSH Terms]) OR ((((computer [Title/Abstract]) OR simulat* [Title/Abstract])) AND ((((model [Title/Abstract]) OR models [Title/Abstract]) OR modelling [Title/Abstract]) OR modeling [Title/Abstract]))	402,382
Filter for the date of interest (by current date)		
3	“1995/01/01” [Date—Publication]: “2017/06/19” [Date—Publication]	16,229,578
Filter with regard to animal population only for exclusion		
4	(Animals [MeSH Terms]) NOT ((Animals [MeSH Terms]) NOT Humans [MeSH Terms])	16,531,079
Types of publications for exclusion		
5	((((((Comment [Publication Type]) OR Letter [Publication Type]) OR “Newspaper Article” [Publication Type]) OR News [Publication Type]) OR Addresses [Publication Type]) OR Editorial [Publication Type]) OR “Published Erratum” [Publication Type]	1,783,994
Combination of search terms		
(((1 AND 2 AND 3) NOT 4) NOT 5)	((((((((((“Diabetes Mellitus” [MeSH Terms]) OR diabet* [Title/Abstract]) OR T2DM [Title/Abstract])) AND (((((((((((markov [Title/Abstract]) OR “monte carlo” [Title/Abstract]) OR “montecarlo” [Title/Abstract]) OR stochastic [Title/Abstract]) OR deterministic [Title/Abstract]) OR “discrete event” [Title/Abstract]) OR “agent based” [Title/Abstract]) OR “agentbased” [Title/Abstract])) OR “Computer Simulation” [MeSH Terms]) OR ((((computer [Title/Abstract]) OR simulat* [Title/Abstract])) AND ((((model [Title/Abstract]) OR models [Title/Abstract]) OR modelling [Title/Abstract]) OR modeling [Title/Abstract]))))) AND “1995/01/01” [Date—Publication]: “2017/06/19” [Date—Publication])) AND ((Animals [MeSH Terms]) NOT ((Animals [MeSH Terms]) NOT Humans [MeSH Terms])))) NOT (((((((Comment [Publication Type]) OR Letter [Publication Type]) OR “Newspaper Article” [Publication Type]) OR News [Publication Type]) OR Addresses [Publication Type]) OR Editorial [Publication Type]) OR “Published Erratum” [Publication Type])	2690

### Technical tools

The references will be collected and stored in the reference manager Mendeley using its cloud-based platform [31]. The abstract and full text screening will be carried out using the web application Rayyan [32]. During the data extraction, we will fill in Google-doc spreadsheet forms that are stored on Google’s cloud storage [33]. All information will be shared and be made available within the working group. The access will be restricted to a private mode.

### Study selection

We will select data studies in several steps. First, we will identify all relevant studies by conducting the search in the scientific publication databases listed in the ‘Information sources’ section. We will remove duplicates to reduce the reviewers’ workload associated with the next steps.

Next, we will screen titles and abstracts of all references selected so far. Two tandems of researchers will independently review and include or exclude references guided by the eligibility criteria mentioned above. The title and abstract screening will be piloted first within the tandems independently and then between the tandems to adjust the procedure. Any disagreement and inconsistency in the final decisions will be recorded and resolved in the further discussion among all researchers involved in the screening.

Full text screening will be the final step within our study selection process. Two independent researchers will review full texts of the studies selected in the previous step to collect the final list of studies for data extraction. The decision on inclusion or exclusion will be made on the basis of the eligibility criteria. The full text screening will be piloted first to adjust the procedure. Any apparent discrepancies appearing during the full text screening will be resolved by a third, independent reviewer.

The screening process will be reported accurately and in sufficient detail—including the title and abstract screening and the full text screening—to complete a PRISMA flowchart [27]. The number of the excluded studies (with reasons for exclusion for those excluded from the full text screening step) will be recorded. Tables for ‘Characteristics of excluded studies’ and ‘Characteristics of included studies’ will be provided.

### Additional data source selection

During the title/abstract screening, we will collect systematic literature reviews addressing diabetes modelling as a potential reference source of interest.

After the full text screening, we will export the reference list of all publications identified for the data extraction. Moreover, we will check forward citations of these publications by tracking them in different citation databases (Scopus, Web of Science). Also, we will apply PubMed’s ‘related articles’ feature for the same papers to collect the first 20 hits. The restricted number of hits is voluntarily chosen to reduce the workload. All references gathered in this step will be subject to the same steps as described in the ‘Study selection’ section.

All researchers who conducted the eligible studies or built the selected models will be contacted by e-mail for information on unpublished or ongoing studies related to the objectives of the systematic review.

We will send a written request to experts in the field through open and private communication channels (i.e. emails, subscription newsletters and professional boards) to check if a pre-final list of the identified models is complete and sufficient. If some undetected models are mentioned, we will include them in our review.

### Data extraction

Two reviewers will independently extract data from all included studies after the full text review by filling in a predefined data extraction spreadsheet. The data extraction spreadsheet was first piloted. The data extraction table is provided in addition to the paper documents (Additional file 2).

Data to be extracted will be arranged into the following categories:

1. Model characteristics;
2. Types of validation involved;
3. External validation: definition, description and use;
4. Data sources used in the external validation: description and use;
5. Results of the external validation: methods and reporting formats;
6. Miscellaneous (conflict of interest and factors not considered).

The data extraction sheet ‘extraction form’ is provided in addition to the paper documents.

Authors of studies included in the full text review that do not provide sufficient information on the external validation in the retrieved articles will be contacted for further information.

### Quality appraisal

The data extraction sheet contains questions and categories that are related to the quality appraisal of the validation. These parameters are based on the collaborative ISPOR, the Academy of Managed Care Pharmacy (AMCP) and the National Pharmaceutical Council (NPC) (ISPOR-AMCP-NPC) guidance on assessing the relevance and credibility of modelling studies [17], the ADA Guidelines for Computer Modelling of Diabetes and Its Complications [9], the Mount Hood Challenge reports [15, 16] and other materials discussing the validation of computer models in health care research. In line with our main objective, the assessment of studies will be limited to the credibility section of the questionnaire. If information is not provided, we will assign ‘Not Applicable’, ‘Not Reported,’ ‘Not Enough Information’ or ‘Not Enough Training’ to the corresponding questions/categories.

### Data synthesis

Since the models are different and the external validation approaches are largely diverse, we will concise results in a narrative descriptive analysis summarizing the definitions and approaches used in different models: data sources, techniques or methods used. In the end, we will provide a summary of what has been done in the external validation of diabetes models up to now.

## Discussion

This protocol describes objectives, methods and steps of an upcoming systematic review that will include studies on external validation of simulation-based computer models designed to represent T2DM incidence and progression in humans. To the best of our knowledge, this contribution to the scientific community is novel since no publications are specially targeting the external validation of T2DM models systematically. The objectives are to identify and define the approaches that are being used for external validation of the T2DM models, appraise the quality of the validation procedures and their reporting and summarize findings to provide a broad overview of the current practice in the field.

### Additional files

Additional file 1: Search submission and peer review assessment, the protocol of peer review of the search terms. The file contains the application and the assessment of the search terms written in MEDLINE syntax and designed for the present review. (PDF 463 kb)

Additional file 2: The data extraction table. The file is an example of a data extraction form that will be used for collecting data in this review. (XLSX 140 kb)

## Appendix

### Details of electronic bibliographic database search strategies

**Table 3:** PRISMA-P (Preferred Reporting Items for Systematic review and Meta-Analysis Protocols) 2015 checklist: recommended items to address in a systematic review protocol* [29]

Section and topic	Item No	Checklist item	PAGE NUMBER (SUBMITTED MANUSCRIPT) AND AUTHOR’S RESPONSE (KO)
ADMINISTRATIVE INFORMATION			
Title:			
Identification	1a	Identify the report as a protocol of a systematic review	See page 1, Title: External validation of type 2 diabetes computer simulation models: definitions, approaches, implications and room for improvement—a protocol for a systematic review
Update	1b	If the protocol is for an update of a previous systematic review, identify as such	Not applicable
Registration	2	If registered, provide the name of the registry (such as PROSPERO) and registration number	See page 2, Section: Systematic review registration: PROSPERO CRD42017069983.
Authors:			
Contact	3a	Provide name, institutional affiliation, e-mail address of all protocol authors; provide physical mailing address of corresponding author	See page 1 Section: Author’s affiliations
Contributions	3b	Describe contributions of protocol authors and identify the guarantor of the review	See page 13, Section: Authors’ contributions
Amendments	4	If the protocol represents an amendment of a previously completed or published protocol, identify as such and list changes; otherwise, state plan for documenting important protocol amendments	See page 13, Section: Protocol amendments
Support:			
Sources	5a	Indicate sources of financial or other support for the review	See page 13, Section: Funding
Sponsor	5b	Provide name for the review funder and/or sponsor	Not applicable
Role of sponsor or funder	5c	Describe roles of funder(s), sponsor(s), and/or institution(s), if any, in developing the protocol	Not applicable
INTRODUCTION			
Rationale	6	Describe the rationale for the review in the context of what is already known	See pages 2 and 4, Section: Background and Study objectives and rationale
Objectives	7	Provide an explicit statement of the question(s) the review will address with reference to participants, interventions, comparators, and outcomes (PICO)	Not applicable
METHODS			
Eligibility criteria	8	Specify the study characteristics (such as PICO, study design, setting, time frame) and report characteristics (such as years considered, language, publication status) to be used as criteria for eligibility for the review	See page 5, Section: Eligibility criteria
Information sources	9	Describe all intended information sources (such as electronic databases, contact with study authors, trial registers or other grey literature sources) with planned dates of coverage	See page 8, Section: Information Sources
Search strategy	10	Present draft of search strategy to be used for at least one electronic database, including planned limits, such that it could be repeated	See Table 2. Search strategy for MEDLINE (via PubMed)
Study records:			
Data management	11a	Describe the mechanism(s) that will be used to manage records and data throughout the review	See page 10, Section: Technical tools
Selection process	11b	State the process that will be used for selecting studies (such as two independent reviewers) through each phase of the review (that is, screening, eligibility and inclusion in meta-analysis)	See page 10, Section: Study selection
Data collection process	11c	Describe planned method of extracting data from reports (such as piloting forms, done independently, in duplicate), any processes for obtaining and confirming data from investigators	See page 11, Section: Data extraction
Data items	12	List and define all variables for which data will be sought (such as PICO items, funding sources), any pre-planned data assumptions and simplifications	See Additional file 2
Outcomes and prioritization	13	List and define all outcomes for which data will be sought, including prioritization of main and additional outcomes, with rationale	Not applicable
Risk of bias in individual studies	14	Describe anticipated methods for assessing risk of bias of individual studies, including whether this will be done at the outcome or study level, or both; state how this information will be used in data synthesis	Not applicable
Data synthesis	15a	Describe criteria under which study data will be quantitatively synthesised	Not applicable
	15b	If data are appropriate for quantitative synthesis, describe planned summary measures, methods of handling data and methods of combining data from studies, including any planned exploration of consistency (such as I^2^, Kendall’s τ)	Not applicable
	15c	Describe any proposed additional analyses (such as sensitivity or subgroup analyses, meta-regression)	Not applicable
	15d	If quantitative synthesis is not appropriate, describe the type of summary planned	See page 12, Section: Data synthesis
Meta-bias(es)	16	Specify any planned assessment of meta-bias(es) (such as publication bias across studies, selective reporting within studies)	Not applicable
Confidence in cumulative evidence	17	Describe how the strength of the body of evidence will be assessed (such as GRADE)	Not applicable

* It is strongly recommended that this checklist be read in conjunction with the PRISMA-P Explanation and Elaboration (cite when available) for important clarification on the items. Amendments to a review protocol should be tracked and dated. The copyright for PRISMA-P (including checklist) is held by the PRISMA-P Group and is distributed under a Creative Commons Attribution Licence 4.0

## Abbreviations

ADA: The American Diabetes Association
AMCP: The Academy of Managed Care Pharmacy
CDC-RTI: The US Centers for Disease Control and Prevention, Research Triangle Institute
CDSR: The Cochrane Database of Systematic Reviews
CENTRAL: Cochrane Central Register of Controlled Trials
CINAHL: Cumulative Index to Nursing and Allied Health Literature
CORE: The Quintiles IMS CORE Diabetes Model
CRC: Centre for Research Communications
DARE: The Database of Abstracts of Reviews of Effects
DM: Diabetes mellitus
EMBASE: Excerpta Medica database
HbA1c: Glycated hemoglobin
IDF: International Diabetes Federation
IFG: Impaired fasting glucose
IGT: Impaired glucose tolerance
INIST/CNRS: Institut de l’information scientifique et technique/Centre national de la recherche scientifique
ISPOR: The International Society for Pharmacoeconomics and Outcomes Research
MEDLINE: Medical Literature Analysis and Retrieval System Online
NHS EED: NHS Economic Evaluation Database
NLM: US National Library of Medicine
NPC: The National Pharmaceutical Council
OpenDOAR: The Directory of Open Access Repositories
OpenGrey: System for Information on Gray Literature in Europe
PQDT: The ProQuest Dissertations & Theses Database
PRESS: Peer Review of Electronic Search Strategies
PRISMA: The Preferred Reporting Items in Systematic Reviews and Meta-analyses
PRISMA-P: The Preferred Reporting Items in Systematic Reviews and Meta-analyses for Protocols
PROSPERO: The International Prospective Register of Systematic Reviews
T1DM: Type 1 diabetes mellitus
T2DM: Type 2 diabetes mellitus
UKPDS: The United Kingdom Prospective Diabetes Study

## References

[1] Ogurtsova K, da Rocha Fernandes JD, Huang Y, Linnenkamp U, Guariguata L, Cho NH (2017). IDF diabetes atlas: global estimates for the prevalence of diabetes for 2015 and 2040. Diabetes Res Clin Pract.

[2] Deshpande AD, Harris-Hayes M, Schootman M (2008). Epidemiology of diabetes and diabetes-related complications. Phys Ther.

[3] The Diabetes Prevention Program Research Group (2007). The prevalence of retinopathy in impaired glucose tolerance and recent-onset diabetes in the diabetes prevention program. Diabet Med.

[4] Porta M, Curletto G, Cipullo D, Rigault de la Longrais R, Trento M, Passera P (2014). Estimating the delay between onset and diagnosis of type 2 diabetes from the time course of retinopathy prevalence. Diabetes Care.

[5] Zhang P, Gregg E (2017). Global economic burden of diabetes and its implications. Lancet Diabetes Endocrinol.

[6] Marshall DA, Burgos-Liz L, IJzerman MJ, Crown W, Padula WV, Wong PK (2015). Selecting a dynamic simulation modeling method for health care delivery research-part 2: report of the ISPOR Dynamic Simulation Modeling Emerging Good Practices Task Force. Value Health.

[7] Briggs ADM, Wolstenholme J, Blakely T, Scarborough P (2016). Choosing an epidemiological model structure for the economic evaluation of non-communicable disease public health interventions. Popul Health Metr.

[8] Salleh S, Thokala P, Brennan A, Hughes R, Booth A. Simulation modelling in healthcare: an umbrella review of systematic literature reviews. PharmacoEconomics. 2017;35(9):937–49. 10.1007/s40273-017-0523-3 28560492

[9] American Diabetes Association Consensus Panel (2004). Guidelines for computer modeling of diabetes and its complications. Diabetes Care.

[10] Lifetime benefits and costs of intensive therapy as practiced in the diabetes control and complications trial. The Diabetes Control and Complications Trial Research Group. JAMA. 1996;276(17):1409–15. Erratum in: JAMA 1997;278(1):25. 8892716

[11] Tarride J-E, Hopkins R, Blackhouse G, Bowen JM, Bischof M, Von Keyserlingk C (2010). A review of methods used in long-term cost-effectiveness models of diabetes mellitus treatment. PharmacoEconomics.

[12] Eastman RC, Javitt JC, Herman WH, Dasbach EJ, Zbrozek AS, Dong F (1997). Model of complications of NIDDM: I. Model construction and assumptions. Diabetes Care.

[13] Yi Y, Philips Z, Bergman G, Burslem K. Economic models in type 2 diabetes. Curr Med Res Opin. 2010;26:2105–18. 10.1185/03007995.2010.494451 20642392

[14] Charokopou M, Sabater FJ, Townsend R, Roudaut M, McEwan P, Verheggen BG (2016). Methods applied in cost-effectiveness models for treatment strategies in type 2 diabetes mellitus and their use in Health Technology Assessments: a systematic review of the literature from 2008 to 2013. Curr Med Res Opin.

[15] Palmer AJ, Hornberger J, Palmer AJ, Mount Hood Modeling Group, Clarke P, Gray A, et al. Computer modeling of diabetes and its complications: a report on the fifth Mount Hood challenge meeting. Value Health 2013;16:453–454. 10.1016/j.jval.2013.04.003 23796276

[16] The Mount Hood 4 Modeling Group (2007). Computer modeling of diabetes and its complications: a report on the fourth Mount Hood challenge meeting. Diabetes Care.

[17] Caro JJ, Eddy DM, Kan H, Kaltz C, Patel B, Eldessouki R (2014). Questionnaire to assess relevance and credibility of modeling studies for informing health care decision making: an ISPOR-AMCP-NPC Good Practice Task Force report. Value Health.

[18] Weinstein MC, O’Brien B, Hornberger J, Jackson J, Johannesson M, McCabe C (2003). Principles of good practice for decision analytic modeling in health-care evaluation: report of the ISPOR task force on good research practices—modeling studies. Value Health.

[19] Eddy DM, Hollingworth W, Caro JJ, Tsevat J, McDonald KM, Wong JB (2012). Model transparency and validation: a report of the ISPOR-SMDM Modeling Good Research Practices Task Force-7. Med Decis Mak.

[20] Brown JB, Palmer AJ, Bisgaard P, Chan W, Pedula K, Russell A (2000). The Mt. Hood challenge: cross-testing two diabetes simulation models. Diabetes Res Clin Pract.

[21] CDC Diabetes Cost-effectiveness Group (2002). Cost-effectiveness of intensive glycemic control, intensified hypertension control, and serum cholesterol level reduction for type 2 diabetes. J Am Med Assoc.

[22] Palmer AJ, Roze SS, Lammert M, Valentine WJ, Minshall ME, Nicklasson L (2004). Comparing the long-term cost-effectiveness of repaglinide plus metformin versus nateglinide plus metformin in type 2 diabetes patients with inadequate glycaemic control: an application of the CORE diabetes model in type 2 diabetes. Curr Med Res Opin.

[23] Eddy DM, Schlessinger L, Kahn R (2005). Clinical outcomes and cost-effectiveness of strategies for managing people at high risk for diabetes. Ann Intern Med.

[24] Becker C, Langer A, Leidl R (2011). The quality of three decision-analytic diabetes models: a systematic health economic assessment. Expert Rev Pharmacoecon Outcomes Res.

[25] Henriksson M, Jindal R, Sternhufvud C, Bergenheim K, Sörstadius E, Willis M (2016). A systematic review of cost-effectiveness models in type 1 diabetes mellitus. PharmacoEconomics.

[26] Kirsch F (2015). A systematic review of Markov models evaluating multicomponent disease management programs in diabetes. Expert Rev Pharmacoecon Outcomes Res.

[27] Liberati A, Altman DG, Tetzlaff J, Mulrow C, Gøtzsche PC, Ioannidis JPA (2009). The PRISMA statement for reporting systematic reviews and meta-analyses of studies that evaluate health care interventions: explanation and elaboration. PLoS Med.

[28] Moher D, Stewart LA, Shekelle P, Ghersi D, Liberati A, Petticrew M (2012). Establishing a new journal for systematic review products. Syst Rev.

[29] Shamseer L, Moher D, Clarke M, Ghersi D, Liberati A, Petticrew M (2015). Preferred reporting items for systematic review and meta-analysis protocols (PRISMA-P) 2015: elaboration and explanation. BMJ.

[30] McGowan J, Sampson M, Salzwedel DM, Cogo E, Foerster V, Lefebvre C (2016). PRESS peer review of electronic search strategies: 2015 guideline statement. J Clin Epidemiol.

[31] Free Reference Manager. https://www.mendeley.com/. Accessed 14 Aug 2017.

[32] Rayyan, the Systematic Reviews web app. https://rayyan.qcri.org/. Accessed 14 Aug 2017.

[33] Google Docs. https://www.google.com/docs/about/. Accessed 14 Aug 2017.

[34] American diabetes association (2012). Diagnosis and classification of diabetes mellitus. Diabetes Care.

[35] Alberti KGMMG, Zimmet PZZ (1998). Definition, diagnosis and classification of diabetes mellitus and its complications. Part 1: diagnosis and classification of diabetes mellitus provisional report of a {WHO} consultation. Diabet Med a J Br Diabet Assoc.

