# Supplementary material for: External validation of type 2 diabetes computer simulation models: definitions, approaches, implications and room for improvement—a protocol for a systematic review
Source: Syst Rev. 2017 Dec 29;6:267. doi: 10.1186/s13643-017-0664-7 (PMC5746956; doi:10.1186/s13643-017-0664-7)
Supplement: Supplementary file 1 — Search submission and peer review assessment, the protocol of peer review of the search terms. The file contains the application and the assessment of the search terms written in MEDLINE syntax and designed for the present review. (PDF 463 kb) [file 13643_2017_664_MOESM1_ESM.pdf]

Appendix A

***PRESS Guideline* — Search Submission & Peer Review Assessment**

SEARCH SUBMISSION: THIS SECTION TO BE FILLED IN BY THE SEARCHER

Searcher: Katherine Ogurtsova	Email: Katherine.Ogurtsova@ddz.uniuesseldorf.de
Date submitted: 07.06.2016	Date requested by: 12.06.2017

Systematic Review Title:

External validation Type 2 diabetes computer simulation models: definitions, approaches, implications and room for improvement.

This search strategy is …

	My PRIMARY (core) database strategy — First time submitting a strategy for search question and database
X	My PRIMARY (core) strategy — Follow-up review NOT the first time submitting a strategy for search question and database. If this is a response to peer review, itemize the changes made to the review suggestions
	SECONDARY search strategy— First time submitting a strategy for search question and database
	SECONDARY search strategy — NOT the first time submitting a strategy for search question and database. If this is a response to peer review, itemize the changes made to the review suggestions

Database

(i.e., MEDLINE,CINAHL…): *[mandatory]*

MEDLINE

Interface

(i.e., Ovid, EBSCO…): *[mandatory]*

PubMed

Research Question

(Describe the purpose of the search) *[mandatory]*

Objectives: to identify and critically appraise approaches that are used for external validation in the existing models of type 2 diabetes development and progression.

Scope of the review:

- computer models of type 2 diabetes incidence and/or progression in human;
- computer models built on simulation techniques on organ/systems, individual or cohort level

PICO Format

(Outline the PICOs for your question — i.e., Patient, Intervention, Comparison, Outcome, and Study Design — as applicable)

**P**	—
**I**	—
**C**	—
**O**	—
**S**	Analysis by using computer simulation model

Inclusion Criteria

(List criteria such as age groups, study designs, etc., to be included) *[optional]*

1. Regarding type 2 diabetes incidence and/or progression (model structure criteria):
   a. Explicit disease state structure: {{healthy} or {IGT} or {IFG} or {pre-diabetes or {high-HbA1c}} and {{type 2 DM} or {type 2 DM with complication(s)}}
   b. Real-life population: a tool must be able to model different populations that vary in age and/or gender structure. The starting population may comprise healthy individual (cohorts) or/and individuals (cohorts) in developed stages of type 2 diabetes with/without complications.
   c. Dynamic projection: a tool must be able to appraise changes in population health over time (both long and short term projections)
   d. Explicit risk-factor states: risk factor states are explicitly modelled. Therefore, the model should account for the risk factor and health status of each simulated individual or subgroup at every time period.
2. Based on simulation techniques
3. Bases on an organ, individual or cohort level simulation
4. Both single- and multi-country studies
5. Studies to be included in the review could be published in English or German language
6. Studies published since 1995

Exclusion Criteria

(List criteria such as study designs, date limits, etc., to be excluded) *[optional]*

1. Editorials, comments, newspaper articles and other forms of popular media;
2. Conference abstracts;
3. Covering only type 1 diabetes;
4. Failure to meet any one of the eligibility criteria above.

Was a search filter applied?

~~Yes~~ No

If YES, which one(s) (e.g., Cochrane RCT filter, PubMed Clinical Queries filter)? Provide the source if this is a published filter. *[mandatory if YES to previous question — textbox]*

Other notes or comments you feel would be useful for the peer reviewer? ***[optional]***

1. I haven't included 'validation' term in the search strategy because I assume that such restriction can be substantially reduce sensitivity of the search strategy since 'validation' may not be reported in the abstracts or it may be performed out of a publication scope or reported later in technical documents or in appendix. Moreover, 'external validation' may only be mentioned in the full text articles and some requests are needed to obtain details.
2. I haven't restricted the search strategy to type 2 diabetes only because a lot of models don't put any emphasis on a distinction between two types.
3. I have truncated the time frame by 1.04.2017 as an example. Later on the time frame will be widened.
4. I've run the search strategy for the relevant papers that were found in the previous literature reviews and/or known in the research field.

Please copy and paste your search strategy here, exactly as run, including the number of hits per line. ***[mandatory]***

N	Short	Search strategy	N of hits
1	1	diabetes[Title/Abstract]	425785
2	2	"1995/01/01"[Date - Publication] : "2017/04/01"[Date - Publication]	16062818
3	3	(Newspaper Article[Publication Type]) OR (News[Publication Type]) OR (Addresses[Publication Type]) OR (Editorial[Publication Type]) OR (Published Erratum[Publication Type])	661743
4	4	animals[MeSH Terms] NOT humans[MeSH Terms]	4329804
5	1 AND 2	(diabetes[Title/Abstract]) AND ("1995/01/01"[Date - Publication] : "2017/04/01"[Date - Publication])	340125
6	5 NOT 3	(((diabetes[Title/Abstract]) AND ("1995/01/01"[Date - Publication] : "2017/04/01"[Date - Publication]))) NOT ((Newspaper Article[Publication Type]) OR (News[Publication Type]) OR (Addresses[Publication Type]) OR (Editorial[Publication Type]) OR (Published Erratum[Publication Type]))	335198
7	6 NOT 4	(((((diabetes[Title/Abstract]) AND ("1995/01/01"[Date - Publication] : "2017/04/01"[Date - Publication]))) NOT ((Newspaper Article[Publication Type]) OR (News[Publication Type]) OR (Addresses[Publication Type]) OR (Editorial[Publication Type]) OR (Published Erratum[Publication Type])))) NOT ((animals[MeSH Terms] NOT humans[MeSH Terms]))	300056

8	8	Markov[Title/Abstract] OR "Monte Carlo"[Title/Abstract] OR stochastic[Title/Abstract] OR deterministic[Title/Abstract] OR "discreet event"[Title/Abstract] OR "agent based"[Title/Abstract] OR ((modelling[Title/Abstract] OR modeling[Title/Abstract] OR model[Title/Abstract]) AND computer[Title/Abstract]) OR ((modelling[Title/Abstract] OR modeling[Title/Abstract] OR model[Title/Abstract]) AND simulat*[Title/Abstract]) OR (“computer simulation”[MeSH Terms])	380256
9	8 AND 7	(((Markov[Title/Abstract] OR "Monte Carlo"[Title/Abstract] OR stochastic[Title/Abstract] OR deterministic[Title/Abstract] OR "discreet event"[Title/Abstract] OR "agent based"[Title/Abstract] OR ((modelling[Title/Abstract] OR modeling[Title/Abstract] OR model[Title/Abstract]) AND computer[Title/Abstract]) OR ((modelling[Title/Abstract] OR modeling[Title/Abstract] OR model[Title/Abstract]) AND simulat*[Title/Abstract]) OR (“computer simulation”[MeSH Terms])))) AND ((((((diabetes[Title/Abstract]) AND ("1995/01/01"[Date - Publication] : "2017/04/01"[Date - Publication]))) NOT ((Newspaper Article[Publication Type]) OR (News[Publication Type]) OR (Addresses[Publication Type]) OR (Editorial[Publication Type]) OR (Published Erratum[Publication Type])))) NOT ((animals[MeSH Terms] NOT humans[MeSH Terms])))	2523

The changes made to the review suggestions:

1. The basic structure of the search strategy has been reconsidered following the reviewer's adapted search. The following changes were made:
    a. The elements from PICO format (Patient, Intervention, Comparison, Outcome, Study Design) were grouped together while limits and filters (publication date range, exclusion of animal studies, and exclusion with regards to selected publication types) were applied to the PICO core on the further steps.
    b. The two broad and relevant MeSH terms, proposed by reviewer, were included. However, instead of MeSH term "Diabetes Mellitus, Type 2", I prefer the broader term "Diabetes Mellitus" Our decision is supported by that fact that in some simulation models [1,2,3] types of the diabetes are not explicitly distinguished or emphasised that might cause a misclassification of a source. I am aware that this also include studies dealing with diabetes type 1 issues that decreases a specificity of a search. However, while truncation "diabet*" is also included in the keyword, a widening of the MeSH term would affect slightly the final number of hits (see line 52 and 53 in the table below).
    c. In the final search strategy, the terms "MODY", "NIDDM" and "T2D" are not used because they have no impact on the number of hits (see line 30 and 31 in the table below) and not improve the specificity of the search (see line 32 in the table below). Moreover, due to novice of these terms, they are rarely used outside of clinical or basic science studies.
2. The new search was checked on a subset of relevant studies and studies mentioned in the previous literature reviews [4, 5, 6, 7, 8, 9]. The new search strategy correctly finds the studies.
3. The following assumptions were used by applying the filters:
    a. Publication date is limited by 1995 on the earliest: The computer simulation of diabetes is a relatively new concept, and the first meeting of health economic simulation modelling groups focusing on diabetes took place only in 1999[[10]] and challenged two simulation models. Two previous systematic literature reviews [4,6] restrict the time frame of a search by 1995. For example, the authors in [6] justified the limitation by the fact that the first publication of a fully integrated, including the full range of complications, economic model of type 1 diabetes mellitus was published in 1996 [[11]]. As shown in other reviews [7], the first influential work on type 2 diabetes modelling was the article "Model of Complications of NIDDM: I. Model construction and assumptions" published in 1997[[12]].
    b. Excluding animals' studies: on purpose to decrease number of hits and increase a specificity of a search. The hedge/filter for humans comes from [13, Chapter 2, section 6.4.11 in Box 6.4.a.]

       in PubMed: NOT (("Animals"[Mesh] NOT ("Animals"[Mesh] AND "Humans"[Mesh]))

In Embase: NOT ([animals]/lim NOT [humans]/lim)
NOT ((exp animal/ or nonhuman/) NOT exp human/)

c. Excluding publication types: on purpose to decrease number of hits and increase a specificity of a search.

The search strategy (updated after the first round of the review):

N	Short	Search strategy	N of hits
		**Subject of interest: Diabetes Mellitus**	
1a		"Diabetes Mellitus, Type 2"[MeSH Terms]	105292
1		Diabetes Mellitus[MeSH Terms]	362913
2		diabet*[Title/Abstract]	518026
3		T2DM[Title/Abstract]	12479
4		T2D[Title/Abstract]	5440
5		NIDDM[Title/Abstract]	6873
6		MODY[Title/Abstract]	1039
7	1 OR 2 OR 3 OR 4 OR 5 OR 6	((((("Diabetes Mellitus"[MeSH Terms]) OR diabet*[Title/Abstract]) OR T2DM[Title/Abstract]) OR T2D[Title/Abstract]) OR NIDDM[Title/Abstract]) OR MODY[Title/Abstract]	572051
8	1 OR 2 OR 3 OR 4	((("Diabetes Mellitus"[MeSH Terms]) OR diabet*[Title/Abstract]) OR T2DM[Title/Abstract]) OR T2D[Title/Abstract]	571905
9	1 OR 2 OR 3	(("Diabetes Mellitus"[MeSH Terms]) OR diabet*[Title/Abstract]) OR T2DM[Title/Abstract]	571849
		**The analysis of interest: Markov process, Monte Carlo**	
10		markov[Title/Abstract]	17204
11		"monte carlo"[Title/Abstract]	40235
12		montecarlo[Title/Abstract]	49
13		stochastic[Title/Abstract]	31279
14		deterministic[Title/Abstract]	11612
15		"discrete event"[Title/Abstract]	813
16		"agent based"[Title/Abstract]	2334
17		agentbased[Title/Abstract]	2
18	11 OR 11 OR 12 OR 13 OR 14 OR 15 OR 16 OR 17	(((((((agentbased[Title/Abstract]) OR "agent based"[Title/Abstract]) OR "discrete event"[Title/Abstract]) OR deterministic[Title/Abstract]) OR stochastic[Title/Abstract]) OR montecarlo[Title/Abstract]) OR "monte carlo"[Title/Abstract]) OR markov[Title/Abstract]	92514
		The analysis of interest: Models and Computer Simulation	
19		"Computer Simulation"[MeSH Terms]	190358
20		computer[Title/Abstract]	190304
21		simulat*[Title/Abstract]	407554
22	20 OR 21	(simulat*[Title/Abstract]) OR computer[Title/Abstract]	562717
23		model[Title/Abstract]	1660447
24		models[Title/Abstract]	726998
25		modelling[Title/Abstract]	57929
26		modeling	181990
27	23 OR 24 OR 25 OR 26	(((modeling) OR modelling[Title/Abstract]) OR models[Title/Abstract]) OR model[Title/Abstract]	2242304
28	22 AND 27	(((simulat*[Title/Abstract]) OR computer[Title/Abstract])) AND ((((modeling) OR modelling[Title/Abstract]) OR models[Title/Abstract]) OR model[Title/Abstract])	204316

N	Short	Search strategy	N of hits
		Analysis of interenst: Computer simulation or Markov process	
29	18 OR 19 OR 28	(((((((((agentbased[Title/Abstract]) OR "agent based"[Title/Abstract]) OR "discrete event"[Title/Abstract]) OR deterministic[Title/Abstract]) OR stochastic[Title/Abstract]) OR montecarlo[Title/Abstract]) OR "monte carlo"[Title/Abstract]) OR markov[Title/Abstract])) OR "Computer Simulation"[MeSH Terms]) OR ((((simulat*[Title/Abstract]) OR computer[Title/Abstract])) AND ((((modeling) OR modelling[Title/Abstract]) OR models[Title/Abstract]) OR model[Title/Abstract]))	397249
30	7 AND 29	((((((("Diabetes Mellitus"[MeSH Terms]) OR diabet*[Title/Abstract]) OR T2DM[Title/Abstract]) OR T2D[Title/Abstract]) OR NIDDM[Title/Abstract]) OR MODY[Title/Abstract]))) AND (((((((((((agentbased[Title/Abstract]) OR "agent based"[Title/Abstract]) OR "discrete event"[Title/Abstract]) OR deterministic[Title/Abstract]) OR stochastic[Title/Abstract]) OR montecarlo[Title/Abstract]) OR "monte carlo"[Title/Abstract]) OR markov[Title/Abstract])) OR "Computer Simulation"[MeSH Terms]) OR ((((simulat*[Title/Abstract]) OR computer[Title/Abstract])) AND ((((modeling) OR modelling[Title/Abstract]) OR models[Title/Abstract]) OR model[Title/Abstract]))))	3817
31	8 AND 29	(((((("Diabetes Mellitus"[MeSH Terms]) OR diabet*[Title/Abstract]) OR T2DM[Title/Abstract]) OR T2D[Title/Abstract]))) AND (((((((((((agentbased[Title/Abstract]) OR "agent based"[Title/Abstract]) OR "discrete event"[Title/Abstract]) OR deterministic[Title/Abstract]) OR stochastic[Title/Abstract]) OR montecarlo[Title/Abstract]) OR "monte carlo"[Title/Abstract]) OR markov[Title/Abstract])) OR "Computer Simulation"[MeSH Terms]) OR ((((simulat*[Title/Abstract]) OR computer[Title/Abstract])) AND ((((modeling) OR modelling[Title/Abstract]) OR models[Title/Abstract]) OR model[Title/Abstract]))))	3817
32	9 AND 29	((((("Diabetes Mellitus"[MeSH Terms]) OR diabet*[Title/Abstract]) OR T2DM[Title/Abstract]))) AND (((((((((((agentbased[Title/Abstract]) OR "agent based"[Title/Abstract]) OR "discrete event"[Title/Abstract]) OR deterministic[Title/Abstract]) OR stochastic[Title/Abstract]) OR montecarlo[Title/Abstract]) OR "monte carlo"[Title/Abstract]) OR markov[Title/Abstract])) OR "Computer Simulation"[MeSH Terms]) OR ((((simulat*[Title/Abstract]) OR computer[Title/Abstract])) AND ((((modeling) OR modelling[Title/Abstract]) OR models[Title/Abstract]) OR model[Title/Abstract]))))	3814
		**Filter for the date of interest**	
33		("1995/01/01"[Date - Publication] : "2017/04/01"[Date - Publication])	16071542
34	32 AND 33	((((((("Diabetes Mellitus"[MeSH Terms]) OR diabet*[Title/Abstract]) OR T2DM[Title/Abstract]))) AND (((((((((((agentbased[Title/Abstract]) OR "agent based"[Title/Abstract]) OR "discrete event"[Title/Abstract]) OR deterministic[Title/Abstract]) OR stochastic[Title/Abstract]) OR montecarlo[Title/Abstract]) OR "monte carlo"[Title/Abstract]) OR markov[Title/Abstract])) OR "Computer Simulation"[MeSH Terms]) OR ((((simulat*[Title/Abstract]) OR computer[Title/Abstract])) AND ((((modeling) OR modelling[Title/Abstract]) OR models[Title/Abstract]) OR model[Title/Abstract])))))) AND ("1995/01/01"[Date - Publication] : "2017/04/01"[Date - Publication])	3575
		**Filter to animal population for excluding**	
35		(Animals[MeSH Terms]) AND Humans[MeSH Terms]	16425808
36		Animals[MeSH Terms]	20760752
37	36 NOT 35	(Animals[MeSH Terms]) NOT ((Animals[MeSH Terms]) AND Humans[MeSH Terms])	4334944

N	Short	Search strategy	N of hits
38	32 NOT 37	(((((((("Diabetes Mellitus"[MeSH Terms]) OR diabet*[Title/Abstract]) OR T2DM[Title/Abstract]))) AND (((((((((((agentbased[Title/Abstract]) OR "agent based"[Title/Abstract]) OR "discrete event"[Title/Abstract]) OR deterministic[Title/Abstract]) OR stochastic[Title/Abstract]) OR montecarlo[Title/Abstract]) OR "monte carlo"[Title/Abstract]) OR markov[Title/Abstract])) OR "Computer Simulation"[MeSH Terms]) OR ((((simulat*[Title/Abstract]) OR computer[Title/Abstract])) AND ((((modeling) OR modelling[Title/Abstract]) OR models[Title/Abstract]) OR model[Title/Abstract])))))) AND ("1995/01/01"[Date - Publication] : "2017/04/01"[Date - Publication]))) NOT ((Animals[MeSH Terms]) NOT ((Animals[MeSH Terms]) AND Humans[MeSH Terms]))	3286
		**Types of publications for exclusion**	
39		Comment[Publication Type]	678629
40		Letter[Publication Type]	951020
41		"Newspaper Article"[Publication Type]	18283
42		News[Publication Type]	181827
43		Addresses[Publication Type]	11228
44		Editorial[Publication Type]	428392
45		"Published Erratum"[Publication Type]	25604
46	39 OR 40 OR 41 OR 42 OR 43 OR 44 OR 45	((((((Comment[Publication Type]) OR Letter[Publication Type]) OR "Newspaper Article"[Publication Type]) OR News[Publication Type]) OR Addresses[Publication Type]) OR Editorial[Publication Type]) OR "Published Erratum"[Publication Type]	1769971
47	38 NOT 46	(((((((((("Diabetes Mellitus"[MeSH Terms]) OR diabet*[Title/Abstract]) OR T2DM[Title/Abstract]))) AND (((((((((((agentbased[Title/Abstract]) OR "agent based"[Title/Abstract]) OR "discrete event"[Title/Abstract]) OR deterministic[Title/Abstract]) OR stochastic[Title/Abstract]) OR montecarlo[Title/Abstract]) OR "monte carlo"[Title/Abstract]) OR markov[Title/Abstract])) OR "Computer Simulation"[MeSH Terms]) OR ((((simulat*[Title/Abstract]) OR computer[Title/Abstract])) AND ((((modeling) OR modelling[Title/Abstract]) OR models[Title/Abstract]) OR model[Title/Abstract])))))) AND ("1995/01/01"[Date - Publication] : "2017/04/01"[Date - Publication]))) NOT ((Animals[MeSH Terms]) NOT ((Animals[MeSH Terms]) AND Humans[MeSH Terms])))) NOT (((((((Comment[Publication Type]) OR Letter[Publication Type]) OR "Newspaper Article"[Publication Type]) OR News[Publication Type]) OR Addresses[Publication Type]) OR Editorial[Publication Type]) OR "Published Erratum"[Publication Type])	3253
48		**Filter for the date of interest (by current date)**	
49		("1995/01/01"[Date - Publication] : "2017/06/06"[Date - Publication])	16226565
50	32 AND 49	((((((("Diabetes Mellitus"[MeSH Terms]) OR diabet*[Title/Abstract]) OR T2DM[Title/Abstract]))) AND (((((((((((agentbased[Title/Abstract]) OR "agent based"[Title/Abstract]) OR "discrete event"[Title/Abstract]) OR deterministic[Title/Abstract]) OR stochastic[Title/Abstract]) OR montecarlo[Title/Abstract]) OR "monte carlo"[Title/Abstract]) OR markov[Title/Abstract])) OR "Computer Simulation"[MeSH Terms]) OR ((((simulat*[Title/Abstract]) OR computer[Title/Abstract])) AND ((((modeling) OR modelling[Title/Abstract]) OR models[Title/Abstract]) OR model[Title/Abstract])))))) AND ("1995/01/01"[Date - Publication] : "2017/06/06"[Date - Publication])	3621

N	Short	Search strategy	N of hits
51	50 NOT 37	(((((((("Diabetes Mellitus"[MeSH Terms]) OR diabet*[Title/Abstract]) OR T2DM[Title/Abstract]))) AND ((((((((((agentbased[Title/Abstract]) OR "agent based"[Title/Abstract]) OR "discrete event"[Title/Abstract]) OR deterministic[Title/Abstract]) OR stochastic[Title/Abstract]) OR montecarlo[Title/Abstract]) OR "monte carlo"[Title/Abstract]) OR markov[Title/Abstract])) OR "Computer Simulation"[MeSH Terms]) OR ((((simulat*[Title/Abstract]) OR computer[Title/Abstract])) AND ((((modeling) OR modelling[Title/Abstract]) OR models[Title/Abstract]) OR model[Title/Abstract])))))) AND ("1995/01/01"[Date - Publication] : "2017/06/06"[Date - Publication]))) NOT ((Animals[MeSH Terms]) NOT ((Animals[MeSH Terms]) AND Humans[MeSH Terms]))	3332
52	51 NOT 46[i]	(((((((((("Diabetes Mellitus, Type 2"[MeSH Terms]) OR diabet*[Title/Abstract]) OR T2DM[Title/Abstract]))) AND ((((((((((agentbased[Title/Abstract]) OR "agent based"[Title/Abstract]) OR "discrete event"[Title/Abstract]) OR deterministic[Title/Abstract]) OR stochastic[Title/Abstract]) OR montecarlo[Title/Abstract]) OR "monte carlo"[Title/Abstract]) OR markov[Title/Abstract])) OR "Computer Simulation"[MeSH Terms]) OR ((((simulat*[Title/Abstract]) OR computer[Title/Abstract])) AND ((((modeling) OR modelling[Title/Abstract]) OR models[Title/Abstract]) OR model[Title/Abstract])))))) AND ("1995/01/01"[Date - Publication] : "2017/06/06"[Date - Publication]))) NOT ((Animals[MeSH Terms]) NOT ((Animals[MeSH Terms]) AND Humans[MeSH Terms])))) NOT ((((((Comment[Publication Type]) OR Letter[Publication Type]) OR "Newspaper Article"[Publication Type]) OR News[Publication Type]) OR Addresses[Publication Type]) OR Editorial[Publication Type]) OR "Published Erratum"[Publication Type])	3159
53	51 NOT 46[ii]	((((((((((("Diabetes Mellitus"[MeSH Terms]) OR diabet*[Title/Abstract]) OR T2DM[Title/Abstract]))) AND ((((((((((agentbased[Title/Abstract]) OR "agent based"[Title/Abstract]) OR "discrete event"[Title/Abstract]) OR deterministic[Title/Abstract]) OR stochastic[Title/Abstract]) OR montecarlo[Title/Abstract]) OR "monte carlo"[Title/Abstract]) OR markov[Title/Abstract])) OR "Computer Simulation"[MeSH Terms]) OR ((((simulat*[Title/Abstract]) OR computer[Title/Abstract])) AND ((((modeling) OR modelling[Title/Abstract]) OR models[Title/Abstract]) OR model[Title/Abstract])))))) AND ("1995/01/01"[Date - Publication] : "2017/06/06"[Date - Publication]))) NOT ((Animals[MeSH Terms]) NOT ((Animals[MeSH Terms]) AND Humans[MeSH Terms])))) NOT ((((((Comment[Publication Type]) OR Letter[Publication Type]) OR "Newspaper Article"[Publication Type]) OR News[Publication Type]) OR Addresses[Publication Type]) OR Editorial[Publication Type]) OR "Published Erratum"[Publication Type]))	3299

[i] The number of hits where "Diabetes Mellitus, Type 2" MeSH term is used.
[ii] The number of hits where broader "Diabetes Mellitus" Mesh term is used.

Bibliography

1. Palmer AJ, Roze SS, Valentine WJ, et al. The CORE Diabetes Model: Projecting long-term clinical outcomes, costs and cost-effectiveness of interventions in diabetes mellitus (types 1 and 2) to support clinical and reimbursement decision-making. Curr Med Res Opin. 2004;20 Suppl 1(s1):S5-26. doi:10.1185/030079904X1980.

2. Gilmer TP, Roze S, Valentine WJ, et al. Cost-effectiveness of diabetes case management for low-income populations. Health Serv Res. 2007;42(5):1943-1959. doi:10.1111/j.1475-6773.2007.00701.x.

3. Mason JM, Freemantle N, Gibson JM, New JP. Specialist Nurse-Led Clinics to Improve Control of Hypertension and Hyperlipidemia in Diabetes: Economic analysis of the SPLINT trial. Diabetes Care. 2004;28(1):40-46. doi:10.2337/diacare.28.1.40.

4. Kirsch F. A systematic review of Markov models evaluating multicomponent disease management programs in diabetes. Expert Rev Pharmacoecon Outcomes Res. 2015;15(6):961-984. doi:10.1586/14737167.2015.1108191.

5. Becker C, Langer A, Leidl R. The quality of three decision-analytic diabetes models: a systematic health economic assessment. Expert Rev Pharmacoecon Outcomes Res. 2011;11(6):751-762. doi:10.1586/erp.11.68.

6. Henriksson M, Jindal R, Sternhufvud C, Bergenheim K, Sörstadius E, Willis M. A Systematic Review of Cost-Effectiveness Models in Type 1 Diabetes Mellitus. Pharmacoeconomics. 2016;34(6):569-585. doi:10.1007/s40273-015-0374-8.

7. Tarride J-E, Hopkins R, Blackhouse G, et al. A Review of Methods Used in Long-Term Cost-Effectiveness Models of Diabetes Mellitus Treatment. Pharmacoeconomics. 2010;28(4):255-277. doi:http://dx.doi.org/10.2165/11531590-000000000-00000.

8. Charokopou M, Sabater FJ, Townsend R, Roudaut M, McEwan P, Verheggen BG. Methods applied in cost-effectiveness models for treatment strategies in type 2 diabetes mellitus and their use in Health Technology Assessments: a systematic review of the literature from 2008 to 2013. Curr Med Res Opin. 2016;32(2):207-218. doi:10.1185/03007995.2015.1102722.

9. Yi Y, Philips Z, Bergman G, Burslem K. Economic models in type 2 diabetes. Curr Med Res Opin. 2010;26(9):2105-2118. doi:10.1185/03007995.2010.494451.

10. Brown JB, Palmer AJ, Bisgaard P, Chan W, Pedula K, Russell A. The Mt. Hood challenge: cross-testing two diabetes simulation models. Diabetes Res Clin Pract. 2000;50:S57-S64. doi:10.1016/S0168-8227(00)00217-5.

11. Lifetime benefits and costs of intensive therapy as practiced in the diabetes control and complications trial. The Diabetes Control and Complications Trial Research Group. JAMA. 1996;276(17):1409-1415. http://www.ncbi.nlm.nih.gov/pubmed/8892716. Accessed June 7, 2017.

12. Eastman RC, Javitt JC, Herman WH, et al. Model of Complications of NIDDM: I. Model construction and assumptions. Diabetes Care. 1997;20(5):725-734.

doi:10.2337/diacare.20.5.725.

13. The Cochrane Collaboration. Cochrane Handbook for Systematic Reviews of Interventions. 5.1.0. (Higgins JPT, Green S, eds.). John Wiley & Sons; 2011. www.handbook.cochrane.org.

PEER REVIEW ASSESSMENT: THIS SECTION TO BE FILLED IN BY THE REVIEWER

Reviewer: Thomas L Heise	Email: heise@bips.uni-bremen.de	Date completed: 02.06.2017

1. TRANSLATION

A ---No revisions	
B --- Revision(s) suggested	X
C --- Revision(s) required	

If "B" or "C," please provide an explanation or example:

N	Short	Search strategy	N of hits
**MeSH term and keywords related to Diabetes Mellitus, Type 2**			
1	1	"Diabetes Mellitus, Type 2"[MeSH]	105135
2	2	diabet*[Title/Abstract]	517783
3	3	T2DM[Title/Abstract]	12466
4	4	T2D[Title/Abstract]	5430
5	5	NIDDM[Title/Abstract]	6873
6	6	MODY[Title/Abstract]	1038
7	1 OR 2 OR 3 OR 4 OR 5 OR 6	("Diabetes Mellitus, Type 2"[Mesh] OR diabet*[Title/Abstract] OR T2DM[Title/Abstract] OR T2D[Title/Abstract] OR "NIDDM"[Title/Abstract] OR "MODY"[Title/Abstract])	528099
**Single keywords related to the analyses of interest**			
8	8	markov[Title/Abstract]	17204
9	9	"monte carlo"[Title/Abstract]	40217[iii]
10	10	"montecarlo"[Title/Abstract]	49
11	11	stochastic[Title/Abstract]	31267
12	12	deterministic[Title/Abstract]	11605
13	13	"discrete event"[Title/Abstract]	813
14	14	"agent based"[Title/Abstract]	2333
15	15	"agentbased"[Title/Abstract]	2
16	8 OR 9 OR 10 OR 11 OR 12 OR 13 OR 14 OR 15	(((((((markov[Title/Abstract]) OR "monte carlo"[Title/Abstract]) OR "montecarlo"[Title/Abstract]) OR stochastic[Title/Abstract]) OR deterministic[Title/Abstract]) OR "discrete event"[Title/Abstract]) OR "agent	92479

[iii] "monte carlo"[Title/Abstract] and "monte-carlo"[Title/Abstract] are retrieving the same records. No variation with "-" is being required.

		based"[Title/Abstract]) OR "agentbased"[Title/Abstract]	
**MeSH term related to the analyses of interest**			
17	17	"Computer Simulation"[MeSH]	190191
**Keyword combinations related to computer- or simulation models**			
18	18	computer[Title/Abstract]	190249
19	19	simulat*[Title/Abstract]	407334
20	18 OR 19	computer[Title/Abstract] OR simulat*[Title/Abstract]	562451
21	21	model[Title/Abstract]	1659645
22	22	models[Title/Abstract]	726602
23	23	modelling[Title/Abstract]	57888
24	24	modeling[Title/Abstract]	175481
25	21 OR 22 OR 23 OR 24	(((model[Title/Abstract]) OR models[Title/Abstract]) OR modelling[Title/Abstract]) OR modeling[Title/Abstract]	2237268
26	20 AND 25	(((computer[Title/Abstract]) OR simulat*[Title/Abstract])) AND ((((model[Title/Abstract]) OR models[Title/Abstract]) OR modelling[Title/Abstract]) OR modeling[Title/Abstract])	203536
**Combination of analyses of interest, including keywords, MeSH, and combinations of keywords**			
27	16 OR 17 OR 26	(((((((((markov[Title/Abstract]) OR "monte carlo"[Title/Abstract]) OR "montecarlo"[Title/Abstract]) OR stochastic[Title/Abstract]) OR deterministic[Title/Abstract]) OR "discrete event"[Title/Abstract]) OR "agent based"[Title/Abstract]) OR "agentbased"[Title/Abstract])) OR “computer simulation”[MeSH]) OR ((((computer[Title/Abstract]) OR simulat*[Title/Abstract])) AND ((((model[Title/Abstract]) OR models[Title/Abstract]) OR modelling[Title/Abstract]) OR modeling[Title/Abstract]))	396676
**Combination of outcome and analyses of interest**			
28	7 AND 27	((("Diabetes Mellitus, Type 2"[Mesh] OR diabet*[Title/Abstract] OR T2DM[Title/Abstract] OR T2D[Title/Abstract] OR "NIDDM"[Title/Abstract] OR "MODY"[Title/Abstract]))) AND ((((((((((markov[Title/Abstract]) OR "monte carlo"[Title/Abstract]) OR "montecarlo"[Title/Abstract]) OR	3630

		stochastic[Title/Abstract]) OR deterministic[Title/Abstract]) OR "discrete event"[Title/Abstract]) OR "agent based"[Title/Abstract]) OR "agentbased"[Title/Abstract])) OR “computer simulation”[MeSH]) OR ((((computer[Title/Abstract]) OR simulat*[Title/Abstract])) AND ((((model[Title/Abstract]) OR models[Title/Abstract]) OR modelling[Title/Abstract]) OR modeling[Title/Abstract])))	
**Publication date of interest**			
29	29	"1995/01/01"[Date - Publication] : "2017/04/01"[Date - Publication]	16070482
**Results limited to publication date of interest**			
30	28 AND 29	((((("Diabetes Mellitus, Type 2"[Mesh] OR diabet*[Title/Abstract] OR T2DM[Title/Abstract] OR T2D[Title/Abstract] OR "NIDDM"[Title/Abstract] OR "MODY"[Title/Abstract]))) AND ((((((((((markov[Title/Abstract]) OR "monte carlo"[Title/Abstract]) OR "montecarlo"[Title/Abstract]) OR stochastic[Title/Abstract]) OR deterministic[Title/Abstract]) OR "discrete event"[Title/Abstract]) OR "agent based"[Title/Abstract]) OR "agentbased"[Title/Abstract])) OR “computer simulation”[MeSH]) OR ((((computer[Title/Abstract]) OR simulat*[Title/Abstract])) AND ((((model[Title/Abstract]) OR models[Title/Abstract]) OR modelling[Title/Abstract]) OR modeling[Title/Abstract]))))) AND "1995/01/01"[Date - Publication] : "2017/04/01"[Date - Publication]	3421
**Filter to animal populations to be facilitated for exclusion**			
31	31	Animals[Mesh] NOT (Humans[Mesh] AND Animals[Mesh])	4333225
**Results with the exclusion of animal studies**			
32	30 NOT 31	((((((("Diabetes Mellitus, Type 2"[Mesh] OR diabet*[Title/Abstract] OR T2DM[Title/Abstract] OR T2D[Title/Abstract] OR "NIDDM"[Title/Abstract] OR "MODY"[Title/Abstract]))) AND ((((((((((markov[Title/Abstract]) OR "monte carlo"[Title/Abstract]) OR "montecarlo"[Title/Abstract]) OR	3141

		stochastic[Title/Abstract]) OR deterministic[Title/Abstract]) OR "discrete event"[Title/Abstract]) OR "agent based"[Title/Abstract]) OR "agentbased"[Title/Abstract])) OR “computer simulation”[MeSH]) OR ((((computer[Title/Abstract]) OR simulat*[Title/Abstract])) AND ((((model[Title/Abstract]) OR models[Title/Abstract]) OR modelling[Title/Abstract]) OR modeling[Title/Abstract]))))) AND "1995/01/01"[Date - Publication] : "2017/04/01"[Date - Publication])) NOT (Animals[Mesh] NOT (Humans[Mesh] AND Animals[Mesh]))	
**Types of publications excluded from the review**			
33	33	Comment[Publication Type]	678522
34	34	Letter[Publication Type]	950809
35	35	"Newspaper Article"[Publication Type]	18283
36	36	News[Publication Type]	181800
37	37	Addresses[Publication Type]	11227
38	38	Editorial[Publication Type]	428230
39	39	"Published Erratum"[Publication Type]	25529
40	33 OR 34 OR 35 OR 36 OR 37 OR 38 OR 39	((((((Comment[Publication Type]) OR Letter[Publication Type]) OR "Newspaper Article"[Publication Type]) OR News[Publication Type]) OR Addresses[Publication Type]) OR Editorial[Publication Type]) OR "Published Erratum"[Publication Type]	1769456
**Results limited to publications of interest**			
41	32 NOT 40	((((((((("Diabetes Mellitus, Type 2"[Mesh] OR diabet*[Title/Abstract] OR T2DM[Title/Abstract] OR T2D[Title/Abstract] OR "NIDDM"[Title/Abstract] OR "MODY"[Title/Abstract]))) AND ((((((((((markov[Title/Abstract]) OR "monte carlo"[Title/Abstract]) OR "montecarlo"[Title/Abstract]) OR stochastic[Title/Abstract]) OR deterministic[Title/Abstract]) OR "discrete event"[Title/Abstract]) OR "agent based"[Title/Abstract]) OR "agentbased"[Title/Abstract])) OR “computer simulation”[MeSH]) OR ((((computer[Title/Abstract]) OR simulat*[Title/Abstract])) AND ((((model[Title/Abstract]) OR models[Title/Abstract]) OR	3113

		modelling[Title/Abstract]) OR modeling[Title/Abstract]))))) AND "1995/01/01"[Date - Publication] : "2017/04/01"[Date - Publication])) NOT (Animals[Mesh] NOT (Humans[Mesh] AND Animals[Mesh])))) NOT (((((((Comment[Publication Type]) OR Letter[Publication Type]) OR "Newspaper Article"[Publication Type]) OR News[Publication Type]) OR Addresses[Publication Type]) OR Editorial[Publication Type]) OR "Published Erratum"[Publication Type])	

Explanation of revision:

The search strategy already incorporates some MeSH and keywords for the "O"utcome of interest, by this, I mean the medical condition (diabetes type 2) and the analyses of interest ("S"). Further incorporation of other elements of the PICO format will likely have no benefit for identifying further studies relevant to the reviews' objective.

However, the following revisions are advised by the reviewer:

- the basic structure of the search strategy should be reconsidered. To improve clarity I highly recommend to distinguish between the elements used from the PICO format and the application of limits and filters (publication date, exclusion of animal studies, and exclusion with regard to selected publication types) (see: adapted search).

2. BOOLEAN AND PROXIMITY OPERATORS

A ---No revisions	
B --- Revision(s) suggested	X
C --- Revision(s) required	

If "B" or "C," please provide an explanation or example:

- boolean operators are being used as intended, this also applies to nesting and brackets.
- the combination of the MeSH and keywords which reflect the concept of diabetes should be connected with "Computer Simulation"[MeSH] in isolation.
- the use of the boolean operator NOT will not likely result in unintended exclusions (e.g. lack of a publication date for a record published after 1995).
- proximity operators are quite limited while searching with the PubMed interface. However, the search is broad enough to account for different scenarios of spelling etc. .

3. SUBJECT HEADINGS

A ---No revisions	
B --- Revision(s) suggested	

C --- Revision(s) required	X

If “B” or “C,” please provide an explanation or example:

- headings are automatically exploded while searching PubMed.
- the updated search strategy includes the two broadest and relevant MeSH terms for the upcoming review.
- without incorporating the MeSH term related to diabetes type 2 some relevant studies might get lost. This also applies for keywords such as “T2DM” where misclassification of MeSH terms might took place.

4. TEXT WORD SEARCHING

A ---No revisions	
B --- Revision(s)suggested	X
C --- Revision(s) required	

If “B” or “C,” please provide an explanation or example:

- the search accounts for various spellings and synonyms (e.g. modeling, modelling etc.).
- truncation of the keyword “diabetes” should be applied (“diabet*”) to account for words such as diabetic etc.. However, this will also include studies dealing with diabetes type 1 issues.
- the newly introduced abbreviations related to the medical concept of diabetes type 2 should be revised by KO before inclusion.
- the limitation of keyword searches to the “Title and Abstract” fields is necessary to limit the search results to a manageable amount, but will likely have no negative impacts on the quality of overall results since the search accounts for different scenarios how simulation models are being introduced to the reader of a reference.

5. SPELLING, SYNTAX, AND LINE NUMBERS

A ---No revisions	
B --- Revision(s)suggested	X
C --- Revision(s) required	

If “B” or “C,” please provide an explanation or example:

- no spelling errors were detected.
- interface specific adaptations of search operators were used correctly.
- all lines included a reference to a search or a result of a combination. However, for the sake of transparency this could be improved (see: adapted search).

6. LIMITS AND FILTERS

A ---No revisions	
B --- Revision(s) suggested	
C --- Revision(s) required	X

If “B” or “C,” please provide an explanation or example:

- the filter to exclude animal studies and the limits with regard to publication date and type should be used at the end of the search to follow a logical order throughout the search syntax.
- relevant filters and limits are correctly adapted to PubMed and reflect the reviews’ objective.
- no sources of the limits and filters used are being cited.
- the publication date limit should be updated before conducting of all database searches will take place.

OVERALL EVALUATION (Note: If one or more “revision required” is noted above, the response below must be “revisions required”.)

A ---No revisions	
B --- Revision(s) suggested	
C --- Revision(s) required	X

Additional comments:

I hope you find my comments useful. Really looking forward to your feedback and this upcoming project. An adapted search accounting for some issues with regard to the search retrieves 1000 additional records. As a consequence, this translates only in a modest increase of workload for the screening purpose.

PEER REVIEW ASSESSMENT: THIS SECTION TO BE FILLED IN BY THE REVIEWER

Reviewer: Thomas L Heise	Email: heise@bips.uni-bremen.de	Date completed: 19.06.2017

1. TRANSLATION

A ---No revisions	X[iv]
B --- Revision(s) suggested	
C --- Revision(s) required	

If "B" or "C," please provide an explanation or example:

N	Short	Search strategy	N of hits
**Subject of interest: Diabetes Mellitus**			
1	1	"Diabetes Mellitus"[MeSH Terms]	363438
2	2	diabet*[Title/Abstract]	519131
3	3	T2DM[Title/Abstract]	12549
4	1 OR 2 OR 3	(("Diabetes Mellitus"[MeSH Terms]) OR diabet*[Title/Abstract]) OR T2DM[Title/Abstract]	572987
**The analysis of interest: Markov process, Monte Carlo**			
5	5	markov[Title/Abstract]	17242
6	6	"monte carlo"[Title/Abstract]	40287
7	7	"montecarlo"[Title/Abstract]	50
8	8	stochastic[Title/Abstract]	31358
9	9	deterministic[Title/Abstract]	11648
10	10	"discrete event"[Title/Abstract]	815
11	11	"agent based"[Title/Abstract]	2349
12	12	"agentbased"[Title/Abstract]	2
13	5 OR 6 OR 7 OR 8 OR 9 OR 10 OR 11 OR 12	(((((((markov[Title/Abstract]) OR "monte carlo"[Title/Abstract]) OR "montecarlo"[Title/Abstract]) OR stochastic[Title/Abstract]) OR deterministic[Title/Abstract]) OR "discrete event"[Title/Abstract]) OR "agent based"[Title/Abstract]) OR "agentbased"[Title/Abstract]	92707
**The analysis of interest: Models and Computer Simulation**			
14	14	"Computer Simulation"[MeSH Terms]	190767
15	15	computer[Title/Abstract]	190533
16	16	simulat*[Title/Abstract]	408585
17	15 OR 16	(computer[Title/Abstract]) OR simulat*[Title/Abstract]	563949
18	18	model[Title/Abstract]	1664480

iv Please see chapter "4. Text word searching" for further instructions.

N	Short	Search strategy	N of hits
19	19	models[Title/Abstract]	729214
20	20	modelling[Title/Abstract]	58112
21	21	modeling[Title/Abstract]	176159
22	18 OR 19 OR 20 OR 21	(((model[Title/Abstract]) OR models[Title/Abstract]) OR modelling[Title/Abstract]) OR modeling[Title/Abstract]	2244199
23	17 AND 22	(((computer[Title/Abstract]) OR simulat*[Title/Abstract])) AND ((((model[Title/Abstract]) OR models[Title/Abstract]) OR modelling[Title/Abstract]) OR modeling[Title/Abstract])	204153[v]
**Analyses of interest: Computer simulation or Markov process**			
24	13 OR 14 OR 23	(((((((((markov[Title/Abstract]) OR "monte carlo"[Title/Abstract]) OR "montecarlo"[Title/Abstract]) OR stochastic[Title/Abstract]) OR deterministic[Title/Abstract]) OR "discrete event"[Title/Abstract]) OR "agent based"[Title/Abstract]) OR "agentbased"[Title/Abstract])) OR "Computer Simulation"[MeSH Terms]) OR ((((computer[Title/Abstract]) OR simulat*[Title/Abstract])) AND ((((model[Title/Abstract]) OR models[Title/Abstract]) OR modelling[Title/Abstract]) OR modeling[Title/Abstract]))	397849
**Combination of subject and analysis of interest**			
25	4 AND 24	(((("Diabetes Mellitus"[MeSH Terms]) OR diabet*[Title/Abstract]) OR T2DM[Title/Abstract])) AND ((((((((((markov[Title/Abstract]) OR "monte carlo"[Title/Abstract]) OR "montecarlo"[Title/Abstract]) OR stochastic[Title/Abstract]) OR deterministic[Title/Abstract]) OR "discrete event"[Title/Abstract]) OR "agent based"[Title/Abstract]) OR "agentbased"[Title/Abstract])) OR "Computer Simulation"[MeSH Terms]) OR ((((computer[Title/Abstract]) OR simulat*[Title/Abstract])) AND ((((model[Title/Abstract]) OR models[Title/Abstract]) OR	3829

[v] The proposed search strategy by the author has no [Title/Abstract] restriction for the keyword „modeling".

N	Short	Search strategy	N of hits
		modelling[Title/Abstract]) OR modeling[Title/Abstract])))	
**Filter for the date of interest (by current date)**			
26	26	"1995/01/01"[Date - Publication] : "2017/06/19"[Date - Publication]	16261812
27	25 AND 26	((((("Diabetes Mellitus"[MeSH Terms]) OR diabet*[Title/Abstract]) OR T2DM[Title/Abstract])) AND (((((((((markov[Title/Abstract]) OR "monte carlo"[Title/Abstract]) OR "montecarlo"[Title/Abstract]) OR stochastic[Title/Abstract]) OR deterministic[Title/Abstract]) OR "discrete event"[Title/Abstract]) OR "agent based"[Title/Abstract]) OR "agentbased"[Title/Abstract])) OR "Computer Simulation"[MeSH Terms]) OR ((((computer[Title/Abstract]) OR simulat*[Title/Abstract])) AND ((((model[Title/Abstract]) OR models[Title/Abstract]) OR modelling[Title/Abstract]) OR modeling[Title/Abstract]))))) AND "1995/01/01"[Date - Publication] : "2017/06/19"[Date - Publication]	3636
**Filter with regard to animal population only for exclusion**			
28		(Animals[MeSH Terms]) AND Humans[MeSH Terms]	16441621
29		Animals[MeSH Terms]	20779718
30	28 NOT 29	(Animals[MeSH Terms]) NOT ((Animals[MeSH Terms]) AND Humans[MeSH Terms])	4338097
31	27 NOT 30	((((((("Diabetes Mellitus"[MeSH Terms]) OR diabet*[Title/Abstract]) OR T2DM[Title/Abstract])) AND ((((((((((markov[Title/Abstract]) OR "monte carlo"[Title/Abstract]) OR "montecarlo"[Title/Abstract]) OR stochastic[Title/Abstract]) OR deterministic[Title/Abstract]) OR "discrete event"[Title/Abstract]) OR "agent based"[Title/Abstract]) OR "agentbased"[Title/Abstract])) OR "Computer Simulation"[MeSH Terms]) OR ((((computer[Title/Abstract]) OR simulat*[Title/Abstract])) AND ((((model[Title/Abstract]) OR models[Title/Abstract]) OR modelling[Title/Abstract]) OR modeling[Title/Abstract]))))) AND "1995/01/01"[Date - Publication] :	3346

N	Short	Search strategy	N of hits
		"2017/06/19"[Date - Publication])) NOT ((Animals[MeSH Terms]) NOT ((Animals[MeSH Terms]) AND Humans[MeSH Terms]))	
**Types of publications for exclusion**			
32	32	Comment[Publication Type]	678827
33	33	Letter[Publication Type]	951898
34	34	"Newspaper Article"[Publication Type]	18283
35	35	News[Publication Type]	181929
36	36	Addresses[Publication Type]	11241
37	37	Editorial[Publication Type]	429314
38	38	"Published Erratum"[Publication Type]	25926
39	32 OR 33 OR 34 OR 35 OR 36 OR 37 OR 38	((((((Comment[Publication Type]) OR Letter[Publication Type]) OR "Newspaper Article"[Publication Type]) OR News[Publication Type]) OR Addresses[Publication Type]) OR Editorial[Publication Type]) OR "Published Erratum"[Publication Type]	1772258
40	31 NOT 39	(((((((((("Diabetes Mellitus"[MeSH Terms]) OR diabet*[Title/Abstract]) OR T2DM[Title/Abstract])) AND ((((((((((markov[Title/Abstract]) OR "monte carlo"[Title/Abstract]) OR "montecarlo"[Title/Abstract]) OR stochastic[Title/Abstract]) OR deterministic[Title/Abstract]) OR "discrete event"[Title/Abstract]) OR "agent based"[Title/Abstract]) OR "agentbased"[Title/Abstract])) OR "Computer Simulation"[MeSH Terms]) OR ((((computer[Title/Abstract]) OR simulat*[Title/Abstract])) AND ((((model[Title/Abstract]) OR models[Title/Abstract]) OR modelling[Title/Abstract]) OR modeling[Title/Abstract]))))) AND "1995/01/01"[Date - Publication] : "2017/06/19"[Date - Publication])) NOT ((Animals[MeSH Terms]) NOT ((Animals[MeSH Terms]) AND Humans[MeSH Terms])))) NOT (((((((Comment[Publication Type]) OR Letter[Publication Type]) OR "Newspaper Article"[Publication Type]) OR News[Publication Type]) OR Addresses[Publication Type]) OR Editorial[Publication Type]) OR "Published Erratum"[Publication Type])	3313

Explanation of minor revision:

- please see chapter “4. Text word searching” for further instructions.
- the revised search strategy definitely improved in terms of readability and clarity (author comment: 1.a.).

2. BOOLEAN AND PROXIMITY OPERATORS

A ---No revisions	X
B --- Revision(s) suggested	
C --- Revision(s) required	

If “B” or “C,” please provide an explanation or example:

- no revisions required.

3. SUBJECT HEADINGS

A ---No revisions	X
B --- Revision(s) suggested	
C --- Revision(s) required	

If “B” or “C,” please provide an explanation or example:

- as suggested two reasonable MeSH terms are now being incorporated in the search strategy. I agree that the broader Diabetes MeSH term makes more sense in order to retrieve relevant diabetes modelling studies (author comment: 1.b.)

4. TEXT WORD SEARCHING

A ---No revisions	
B --- Revision(s)suggested	
C --- Revision(s) required	X

If “B” or “C,” please provide an explanation or example:

- the exclusion of suggested keywords (“MODY”, “NIDDM” and “T2D”) seems reasonable to me based on the author’s statement. I also checked the impact and found no reasons why the key words should be included. However, that might change for updates of this review in the future (author comment: 1.c.).
- the proposed search strategy has no [Title/Abstract] restriction for the keyword „modeling“. Please change this in order to streamline your search approach with regard to database fields (first occurrence in line 28 onwards based on the revised search of the author).

5. SPELLING, SYNTAX, AND LINE NUMBERS

A ---No revisions	X

B --- Revision(s)suggested	
C --- Revision(s) required	

If "B" or "C," please provide an explanation or example:

- no spelling errors were detected.
- interface specific adaptations of search operators were used correctly.
- all lines included a reference to a search or a result of a combination.
- no revisions required.

6. LIMITS AND FILTERS

A ---No revisions	X
B --- Revision(s) suggested	
C --- Revision(s) required	

If "B" or "C," please provide an explanation or example:

- citations of limits and filters were updated (author comment: 3.).
- the publication date limit should be updated before conducting the search.
- no revisions required.

OVERALL EVALUATION (Note: If one or more "revision required" is noted above, the response below must be "revisions required".)

A ---No revisions	
B --- Revision(s) suggested	
C --- Revision(s) required	X

Additional comments:

The overall search structure is now very clear to me and the upcoming results of the search should serve as a solid reference base. However, the author is advised to consider the minor flaw with regard to chapter "4. Text word searching". Keeping this in mind, no further revisions are required. Please see the revised search strategy - in green colour - with adjusted search hits. Looking forward to this review.
